# Supplementary material for: A qualitative examination of the factors affecting the adoption of injury focused wearable technologies in recreational runners
Source: PLoS One. 2022 Jul 6;17(7):e0265475. doi: 10.1371/journal.pone.0265475 (PMC9258862; doi:10.1371/journal.pone.0265475)
Supplement: S2 Table — (DOCX) [file pone.0265475.s002.docx]

**S2 Table: Focus group introduction, aims, schedule domains, and sample questions**

| **Domain** | **Sample questions** |
| --- | --- |
| Sample brief introduction/Aims of study | Hi everyone. I am conducting research on the use of wearable technologies to monitor running-related injuries. The aims of this focus group are to gather your thoughts on the important metrics to monitor for running-related injuries using wearable technologies. We will also have a discussion on injury focused technologies and why you would or would not use them. If you have any questions at any point, please let me know. |
| Conversation openers | Can you tell me about the types of technologies you use while running? |
| Perceived barriers to the use of injury-focused technologies | Would anything discourage you from engaging with an injury-focused application? |
|  | Would anything discourage you from wearing an injury-focused device or sensor? |
| Perceived facilitators to the use of injury-focused technologies | Would anything encourage you to engage with an injury-focused application? |
|  | Would anything encourage you to wear an injury-focused device or sensor? |
| Metrics perceived as important for monitoring RRI risk | What are the risk factors for injury that you think should be monitored with wearable technologies? |
|  |  |
|  |  |
