# Supplementary material for: A qualitative examination of the factors affecting the adoption of injury focused wearable technologies in recreational runners
Source: PLoS One. 2022 Jul 6;17(7):e0265475. doi: 10.1371/journal.pone.0265475 (PMC9258862; doi:10.1371/journal.pone.0265475)
Supplement: S3 Table — (DOCX) [file pone.0265475.s003.docx]

**S3 Table: Coding framework**

| 1. Metrics perceived as important to monitor for injury risk | | | | |
| --- | --- | --- | --- | --- |
| **Core categories** | **Themes** | **Sub-themes** | | |
| 1.1 Overtraining | 1.1.1 Excessive loading | 1.1.1.1 High accumulative load | | |
|  |  | 1.1.1.2 High intensity training | | |
|  |  | 1.1.1.3 In-session fatigue | | |
|  |  | 1.1.1.4 Lower running experience | | |
|  | 1.1.2 Inadequate recovery | 1.1.2.1 Fatigue and poor sleep | | |
|  |  | 1.1.2.2 Insufficient rest days | | |
|  |  | 1.1.2.3 Poor nutrition | | |
|  |  | 1.1.2.4 High stress | | |
| 1.2 Training-related risk factors | 1.2.1 Running environment | 1.2.1.1 Terrain | | |
|  |  | 1.2.1.2 Weather | | |
|  | 1.2.2 Training activities | 1.2.2.1 Current training activities | | |
|  |  | 1.2.2.2 Historic training activities | | |
|  | 1.2.3 Running technique | 1.2.3.1 Foot strike | | |
|  |  | 1.2.3.2 Cadence | | |
|  |  | 1.2.3.3 Bilateral asymmetry | | |
|  | 1.2.4 Footwear | 1.2.4.1 Infrequent changing of footwear | | |
|  |  | 1.2.4.2 Type of footwear | | |
| 1.3 Individual related risk factors | 1.3.1 Injury history | 1.3.1.1 Ongoing niggle | | |
|  |  | 1.3.1.2 Previous injury | | |
|  | 1.3.2 Demographic information | 1.3.2.1 Age | | |
|  |  | - - - 1. Body mass index | | |
|  |  | - - - 1. Sub-optimal biomechanics | | |
|  | 1.3.3 Type of runner | - - - 1. Preferred distance/event | | |
|  | 1.3.4 Psychological parameters | - - - 1. Mood | | |
|  |  | 1.3.4.2 Perception of run | | |
|  |  | 1.3.4.3 Psychological readiness to run | | |
| 1. Barriers to the use of injury focused running technologies | | | | |
| **Core categories** | **Themes** | **Sub-themes** | **Secondary sub-themes** | **Tertiary sub-themes** |
| 2.1 Difficult to use | 2.1.1 Device design | 2.1.1.1 Specifications of device | 2.1.1.1.1 Bulky | |
|  |  |  | 2.1.1.1.2 Large | |
|  |  | 2.1.1.2 Application method | 2.1.1.2.1 Time consuming set up | |
|  |  |  | 2.1.1.2.2 Adapting clothing/Extra clothing | |
|  |  |  | 2.1.1.2.3 Belt mechanism | 2.1.1.2.3a Irritating/Uncomfortable |
|  |  |  |  | 2.1.1.2.3b Not secure |
|  |  | 2.1.1.3 Location | 2.1.1.3.1 Lower back/Waist | 2.1.1.3.1a Uncomfortable/ Irritating |
|  |  |  |  | 2.1.1.3.1b Not secure |
|  |  |  | 2.1.1.3.2 Uncomfortable/Irritating (non-specific location) | |
|  |  |  | 2.1.1.3.3Wrist/Arm | 2.1.1.3.3a Uncomfortable/ Irritating |
|  |  |  |  | 2.1.1.3.3b Not secure |
|  |  |  | 2.1.1.3.4 Obvious/Noticeable to others (non-specific location) | |
|  |  |  | 2.1.1.3.4 Foot/Shoe | 2.1.1.3.4a Inconvenient |
|  |  |  | 2.1.1.3.5 Chest/Torso | 2.1.1.3.5a Uncomfortable/ Irritating |
|  |  |  |  | 2.1.1.3.5b Not secure |
|  |  | 2.1.1.4 Technical issues | 2.1.1.4a Frequent charging of device | |
|  |  |  | 2.1.1.4b Bluetooth connection issues | |
|  |  |  | 2.1.1.4c Broken device | |
|  |  |  | 2.1.1.4d Unclean device | |
|  | 2.1.2 Application design | 2.1.2.1 Data input | 2.1.2.1a Time consuming | |
|  |  |  | 2.1.2.1b High quantity of questions | |
|  |  |  | 2.1.2.1c Repetitive/Irrelevant data required | |
|  |  |  | 2.1.2.1d High text input | |
|  |  | 2.1.2.2 Data use | 2.1.2.2a Ambiguity of data use | |
| 2.2 Feedback | 2.2.1 Irrelevant feedback |  |  | |
|  | 2.2.2 Too much data |  |  | |
|  | 2.2.2 Inaccurate feedback |  |  | |
|  | 2.2.3 Feedback delivery | - - - 1. Email |  | |
| 1. Facilitators to the use of injury-focused running technologies | | | | |
| 3.1 Ease of use | 3.1.1 Application design | 3.1.1.1 User-friendly system | 3.1.1.1a Quick input session | |
|  |  |  | 3.1.1.1b Multiple choice questions | |
|  |  |  | 3.1.1.1c Synced with other applications/devices | |
|  |  |  | 3.1.1.1d Notification reminders | |
|  |  |  | 3.1.1.1e Automatic downloading of data from sensor | |
|  |  | 3.1.1.2 Current usage habits | 3.1.1.2a Fits with current usage habits | |
|  | 3.1.2 Device design | 3.1.2.1 Application method | 3.1.2.1.1 Comfortable (non-specific application method) | |
|  |  |  | 3.1.2.1.2 Discrete (non-specific application method) | |
|  |  |  | 3.1.2.1.3 Convenient (non-specific application method) | |
|  |  |  | 3.1.2.1.4 Belt mechanism | 3.1.2.1.4a Convenient |
|  |  |  |  | 3.1.2.1.4b Stable |
|  |  |  | 3.1.2.1.5 Clip mechanism | 3.1.2.1.5a Convenient |
|  |  | 3.1.2.2 Location | 3.1.2.2.1 Lower back/Waist | 3.1.2.2.1a Convenient |
|  |  |  |  | 3.1.2.2.1b Discrete |
|  |  |  |  | 3.1.2.2.1c Comfortable |
|  |  |  |  | 3.1.2.2.1d Stable |
|  |  |  | 3.1.2.2.2 Wrist/Arm | 3.1.2.2.2a Convenient |
|  |  |  |  | 3.1.2.2.2b Stable |
|  |  |  | 3.1.2.2.3 Chest/Torso | 3.1.2.2.3a Convenient |
|  |  |  |  | 3.1.2.2.3b Stable |
|  |  |  | 3.1.2.2.4 Foot/Shoe | 3.1.2.2.4a Convenient |
|  |  |  |  | 3.1.2.2.4b Stable |
|  |  |  | 3.1.2.2.5 Ankle | 3.1.2.2.5a Convenient |
|  |  |  |  | 3.1.2.2.5b Discrete |
|  |  |  | 3.1.2.2.6 Thigh | 3.1.2.2.6a Thigh |
|  |  | 3.1.2.3 Specifications of sensor | 3.1.2.3a Small |  |
|  |  |  | 3.1.2.3b Lightweight |  |
|  |  | 3.1.2.4 Good technical features | 3.1.2.4a Infrequent charging of device | |
|  |  |  | 3.1.2.4b Strong Bluetooth connection | |
| 3.2 Feedback received | 3.2.1 Injury-related feedback | 3.2.1.1 Reduce injury risk |  |  |
|  |  | 3.2.1.2 Understand injury mechanisms |  |  |
|  |  | 3.2.1.3 Monitor rehabilitation from injury |  |  |
|  |  | 3.2.1.4 Advice/Recommendations |  |  |
|  |  | 3.2.1.5 Comparison to cohort |  |  |
|  |  | 3.2.1.6 Extend running career |  |  |
|  | 3.2.2 Enhanced data  4 focus groups | 3.2.2.1 Performance insights | 3.2.2.1a Performance progressions | |
|  |  |  | 3.2.2.1b Optimizing performance | |
|  |  | 3.2.2.2 Additional data | 3.2.2.1a Cadence/Stride information | |
|  |  |  | 3.2.2.1b Technique | |
|  |  |  | 3.2.2.1c Power | |
|  |  |  | 3.2.2.1d Comparison to cohort | |
|  |  |  | 3.2.2.1feMonitor recovery from training | |
|  | 3.2.3 Feedback delivery | 3.2.3.1 Choice of feedback delivery |  | |
|  |  | 3.2.3.2WhatsApp/Text |  | |
