# Supplementary material for: A qualitative examination of the factors affecting the adoption of injury focused wearable technologies in recreational runners
Source: PLoS One. 2022 Jul 6;17(7):e0265475. doi: 10.1371/journal.pone.0265475 (PMC9258862; doi:10.1371/journal.pone.0265475)
Supplement: S4 Table — (DOCX) [file pone.0265475.s004.docx]

**S4 Table: Supplementary quotes**

Supplementary quotes of running-related injury risk factors perceived as important to monitor using wearable technology devices by recreational runners

| **Theme** | **Sub-theme** | **Exemplary quotes** |
| --- | --- | --- |
| Excessive loading | High accumulative load | M1: *“the single biggest injury I had was the stress fracture. And the cause of that is load… if you're going from 20 miles one week to 50 miles the next week, and yoyo-ing like that constantly… the kind of loads that that places on the body, especially… if you're including in that speed work, is inevitably going to lead to injury”* |
|  | High intensity training | F2: *“a lot of people don't leave a gap between their hard runs and you're not getting the… difference… Every run shouldn't be the same… In terms of injury… if you're doing three hard runs in a row and just doing one long easy one, then maybe your training isn't improving and… the injury could be getting into it”* |
|  | In-session fatigue | M7: *“... The more tired I get, the heart rate goes up, and if I try and stick to a particular pace, the whole form goes out. And for me, I would think that would lead to more injuries in that regard....Particularly on a long run, I suppose everybody notices that you start to slump that bit more, the form goes… and that's one thing I’m very, very wary of”* |
|  | Less running experience | M9: *“somebody maybe not that experienced reads something decides to change how they run… and then that leads to an injury. As opposed to a more experienced runner who will… be very careful about things that they change and are less likely to get injured”* |
| Inadequate recovery | Fatigue & poor sleep | F4: *“I just think fatigue sometimes too… like you're not sleeping properly”* |
|  | Poor nutrition | M1: *“I’m always interested in how nutrition and iron and that kind of thing affect the body, and how we look after ourselves. You know, we forget that there's a huge amount of work and there's a huge amount of energy involved in running. And we don't fuel ourselves and look after ourselves in general. You learn too late that you’ve probably done damage already”* |
|  | Insufficient rest days | F3: *“if you're over running? Because there's a lot of people that… probably are injured because they're not actually taking rest days and they’re… going out every day”* |
|  | High stress | F12: *“my Garmin probably has this kind of thing on it… stress levels or how much sleep you got… Because I find, I don’t know whether it’s the PhD, I find like my recovery’s really slow at the moment, which probably isn't good for injuries. So I'd be really interested in seeing… if you’re injured when I’m not sleeping well, when I’m stressed about other things”* |
| Training activities | Concurrent training activities | M2: *“very important to take into account what other sports they’re doing… If you're playing golf you're obviously predisposed... to knee problems and lower back problems… If you’re doing other track events, like javelin or shot put or something, it’s different rotational movements. So I think important to take into account or to track other sporting activity to see if it's an injury related to running, versus... related to something else, or the compound of both”* |
|  | Previous training activities | M9: “*... we were saying that people that had taken up running later in life, without doing any major sports before that, tended to have less injuries. Whereas people like us, that played… soccer, Gaelic, whatever… tended to pick up more injuries because they were running after having all these injuries in the other sports as well… I think our history is… a little bit of a factor as well…. all my problems are as a result of injuries from a previous sport… whereas other people will have a sort of blank slate coming into it, and you might say ‘oh God… they must be doing everything right because they're not getting injured’, ehm, but they also haven’t put their body through… 10 years of hard slog in another sport as well”* |
| Running technique | Foot strike | M13: *“If you could see some… asymmetry in the heel strike or ground contact time… one side or the other... Like vertical oscillation or like, some sort of orientation of the feet, you know, or pelvis or hips, with respect to one another as you move”* |
|  | Cadence | M1: *“... to understand why maybe a foot strike issue or an alignment issue might be coming in… your cadence and the amount of which your foot strike goes out of sync as you go into the longer distances… Your cadence rate drops or increases to try and keep with a pace, when your foot strike is wrong… when you’re trying to sprint and over-stride”* |
|  | Bilateral limb differences | M7: *“whether you’re landing heavier left or right foot… I’m sort of conscious of that… I have a raised arch on my right foot… so that leads to me having to occasionally be wary of that… I see if I notice that I'm tending to favour… my left over my… right. That means I probably heading towards something going wrong with the right foot”* |
| Running environment | Terrain | F12: *“I actually thought of this because I have a little injury at the moment, it's like a flare up of an old injury…. anecdotally, everyone will be like ‘you know concrete is really bad’. Because I found… I did like a race thing on concrete, and I wouldn’t normally run on concrete, but I don’t know if it was the race, or if it was the concrete, but I have like a flare up from an injury, from that”* |
|  | Weather | M4: *“I think maybe the temperature of the run. Because on a… cold day you might be more susceptible to getting injured than a hot day”* |
| Footwear | Infrequent changing of footwear | F11: *“... footwear. I feel like so many people don't change their runners often enough and I really think that's a huge factor in injuries… some people only change their runners every year, and they might be running every day”* |
|  | Type of footwear | M10: *“... what runners people wear… I’d kind of be particular with what kind of runners I wear… spikes for the track, or flats…if I was running a race I’d be wearing… Vaporfly’s… if I was running on wet ground I’d wear my trail runners… taking into account what runners people wear”* |
| Injury status & history | Ongoing niggle | F1: *“... say if you're after coming back from running and you're like ‘oh my knee is a little bit sore’. You're not injured, but there could be little tweaks that may lead to other injuries… like there's always something sore after a run, so it’s tracking the little niggles that might actually lead to something”*  F2: “*… And we don't always remember those little niggles”*  F1: *“… until you're halfway through the next run and you're like ‘Oh, this is sore again remember this was sore”*  M7: *I’d be intrigued to know… all of us have the little niggles and aches and stuff like that… are they precursors to an injury or are they just the little aches and pains that we all get? Because I tend to, if I get an ache or pain or a niggle, I tend not to ignore it and I wait until it goes away… but by virtue of the fact that I quit when I do, is that what has kept me injury free? As opposed to, some people tend to run through these things, they’ll run it off. I keep hearing that expression from other people, ‘actually you can run that off’, or something like that… I’d be intrigued to know is that the niggles that all of us have, can you ignore them as opposed to them turning into an injury”* |
|  | Previous injury | M9: *“The chronic injuries I have, more or less, have been there since before I took up running… I actually don't get a lot of injuries from running, it's more about trying to manage the old ones… I've actually had very, very few running injuries, but all my problems are as a result of injuries from a previous sport”* |
| Population characteristics | Age | M8: “when you're getting older, you’re probably going to get more injury prone as well” |
|  | Body mass index | M8: “the more you weigh I'd say that the higher your impact forces, and I guess that will be at a straight impact.. on the risk factors” |
|  | Sub-optimal biomechanics | M7: *“I have a natural claw foot… I have a raised arch on my right foot… so that leads to me having to occasionally be wary of that”* |
| Type of runner | Preferred distance/event | M4: *“the type of runner… is it a hill runner or is it a 5km park runner, a very fast 5km park runner, or a marathon runner, or a 10 km runner. Because I presume all those different types, would have different injuries and susceptibility to injury as well”* |
| Psychological parameters | Mood | F5: *“... motivation levels on a day. Because some days I’ll go out and I’ll be like really energized for a run,*  *and some days I’d be like ‘oh I don’t want to run’, but I know I have to run. So you run the 5km both days, but like if you're not motivated, you might not be watching the way you run, or you might not be taking care of a pre-existing injury”* |
|  | Perception of run | F14: *“how the run felt, like exertion and then fatigue in general… like the pace in terms of how they're feeling. Was it a really hard run”* |
|  | Psychological readiness to run | F5: *“... mental tiredness… if you have a heavy day at work”* |

Supplementary quotes of perceived barriers to the use of injury-focused running technologies

| **Sub-theme** | **Secondary sub-theme** | **Exemplary quotes** |
| --- | --- | --- |
| Attachment method | Time consuming | F11: *“I wouldn't want it to take too long to have to set up and get in place… It might kind of put you off actually going on the run… I think if you’re doing that every run you went on, you kind of get fed up of it fairly quickly”* |
|  | Adapting/Additional clothing required | M8*: “If I need to wear some contraption or something, I probably wouldn't be much in favour of it… As long as it doesn't rub… that’s the main thing… if it’s rubbing against your skin and getting a bit sore or something like that, that would be my, my issue”* |
|  | Belt mechanism | M1: *“anything like a… belt. You see people wearing belts with water holders and stuff like that… in particular for marathons and longer runs, I've always struggled with those. I find them uncomfortable, and the wearing for the most part, unnecessary”* |
| Technical issues | Frequent charging | F3: *“The charging will be important, because I find… with the watch, you know, it can put me off, like if the battery is low on it… you need it to be easy to charge and that it doesn’t need to be charged too frequently”* |
|  | Bluetooth connection issues | F3: *“ I have earphones that are wireless… they do disconnect quite frequently which can be quite frustrating during the middle of a run… just to try and make sure that whatever way that that’s set up right”* |
|  | Broken device | F11: *“it just depends how sturdy they are, but I could definitely imagine… one falling off during a run and me like breaking it or something”* |
|  | Unclean device | F11: *“how to clean them… nobody wants to be putting on the same sweaty sensor”* |
| User input requirement | Time consuming | F11: *“say you're going on a run like three times a week and you just kind of, input it straight after it, and then it’s kind of done. Once… you don't have to be going back… later that night… I think once you can… do your run and do the app, then you'd be fine… Just kind of get it over and done with… Once you don't have to be spending too much time”* |
|  | High quantity of questions | M12: *“things that would put me off would be off, asking for the whole session… exercises from 12345… And then rest periods or tempos… because that would just be a bit tedious”* |
|  | Repetitive/Irrelevant data required | M11: *“It just gets a bit tedious… basically it’d ask you loads of questions, and it's like the same questions over and over”* |
|  | High text input requirement | M8: *“if you had to write half an essay every time, I think that gets old very quickly”* |
| Data use | Ambiguity of data use | F8: *“be very clear on the data and what the data is being used for… people… can be a little bit funny about… where the data is actually being used. So just that it's very clear that whatever they're signing up for they know where the data is going to be used”* |
| Attachment method | Uncomfortable/Irritating | M8: *“if it’s rubbing against your skin and getting a bit sore or something… that would be my issue… that would be my main concern, if it starts rubbing against your skin and the skin gets rubbed, then that's an issue”* |
|  | Time consuming | F11: *“I wouldn't want it to take too long to have to set up and get in place… It might kind of put you off actually going on the run… I think if you’re doing that every run you went on, you kind of get fed up of it fairly quickly”* |
|  | Adapting/Additional clothing required | M8*: “If I need to wear some contraption or something, I probably wouldn't be much in favour of it… As long as it doesn't rub… that’s the main thing… if it’s rubbing against your skin and getting a bit sore or something like that, that would be my, my issue”* |
|  | Belt mechanism | M1: *“anything like a… belt. You see people wearing belts with water holders and stuff like that… in particular for marathons and longer runs, I've always struggled with those. I find them uncomfortable, and the wearing for the most part, unnecessary”* |
| Location | Low back | M2: *“I've had those fuel belts for the marathons… you just can't wait to throw it away… just gets annoying after a while”* |
|  | Wrist/Arm | M3: *“I'm not a big fan of the… arm monitors… but I haven't used one for a while and I don't know how big this is”* |
|  | Obvious/Noticeable to others | F11: *“I also wouldn't like it if was very obvious…That I was going by with this… fluorescent thing hanging around my waist, and people are like ‘what is that?’”* |
|  | Foot/Shoe | M8: *“If it's a sensor on the shoe, like if you have to tie your shoelaces it's a bit awkward because… I rotate my shoes, and I would have to change it every single time I go running”* |
|  | Chest/Torso | M4: *“I definitely wouldn't like one strapped around the chest… I got a heart rate monitor before with the Garmin and I just couldn't run with it. So I ran about twice with it and it’s in a press ever since”* |
| Specifications of device | Bulky | F9: *“something that's not too heavy or not too bulky or something, that, that it would impact their running”* |
|  | Large | M1: *“if it's something larger than mobile phones…that's… different”* |
| Technical issues | Frequent charging | F3: *“The charging will be important, because I find… with the watch, you know, it can put me off, like if the battery is low on it… you need it to be easy to charge and that it doesn’t need to be charged too frequently”* |
|  | Bluetooth connection issues | F3: *“ I have earphones that are wireless… they do disconnect quite frequently which can be quite frustrating during the middle of a run… just to try and make sure that whatever way that that’s set up right”* |
|  | Broken device | F11: *“it just depends how sturdy they are, but I could definitely imagine… one falling off during a run and me like breaking it or something”* |
|  | Unclean device | F11: *“how to clean them… nobody wants to be putting on the same sweaty sensor”* |
| Useless feedback | Irrelevant feedback | M11: *“when I was using the Whoop… I stopped using it because… it tells you recovery scores, and all this sort of thing… You’d wake up in the morning and it would tell you how you're feeling, and then that would… determine how you felt… I just stopped using it because… I don’t really want to know if I got bad sleep, because… I feel like I had bad sleep all day”* |
|  | Too much feedback | M13: *“all these diet apps, that are… tracking your calories in and calories out… there's just too much in those. And I'm like ‘oh God, I can't use this this, this is annoying me’”* |
|  | Inaccurate feedback | F2: *“I was getting rid of the watch when I was injured or not running well so… you're not becoming consumed by the data like you do with the watches”* |
|  | Feedback delivery | F14: *“I get lost in the amount of emails I get from college, and on top of that you have your private one then… I see emails all the time and I’m just like ‘ugh, what is this now?’... it’s nearly like a negative thing attached with emails”* |

Supplementary quotes of perceived facilitators to the use of injury-focused technologies

| **Sub-theme** | **Secondary sub-theme** | **Exemplary quotes** |
| --- | --- | --- |
| User friendly system | Quick input session | F8: *“I think once it wasn't too onerous… if it was just like asking for three or four questions… that are related to the injury then I certainly wouldn't see an issue with it… once it's very user friendly and that it doesn’t take a lot of time”* |
|  | Multiple choice questions | M9: *“... I’d be more encouraged to do it if there's a lot more, you know, tick the box, rate the scale type things, as opposed to having to type in on your phone… Just tick the box or rate it one to five, as opposed to having to write in stuff”* |
|  | Synced with other applications/devices | F9: *“If you could connect to some of the other Apps that we’re using, like the Garmin one or something for your sleep. Because the Garmin can track your sleep, or if you're using, like My Fitness Pal, if you were inputting your data for… what you’re eating there, it'd be very handy, because then you can just go straight across like… it has all the information”* |
|  | Notification reminders | F9: *“a reminder as well to do it, like a notification coming up… is really handy, because it's easy forget”* |
|  | Automatic downloading of data from device | M5: *“I suppose that the less that data we have to put in, the better”*  *…*  M3: *“I think you're right M5, especially if the information is already there, maybe you can get it from Strava and tie it in”* |
| Current habits | Fits with current usage habits | F14: *“I suppose it is a good window because you're looking at Strava, you’re looking at what you're doing, so you're kind of in the running zone… after your run could be a good time to input some data.. I definitely would have the time then”* |
| Location | Low back/Waist | F1: *“I have one of them now and it just has one zip on it, and if I’m going for… a long run, I put my phone in it… my phone sits… on my lower back. And it doesn’t bother me at all”* |
|  | Wrist/Arm | M11: *“I’ve also used… a wristband… and… you just don't notice that… So the wrist isn't a bad spot for an extra monitor”* |
|  | Chest/Torso | M2: *“the chest strap obviously is very fine, so if it could be integrated into the chest strap… no problem”* |
|  | Foot/Shoe | M9: *“I'd actually... be much more likely to use the one on the shoe. Because… sometimes I'm running from home, sometimes I'm running for work, sometimes I'm running from the gym… sometimes I have a bag with me, sometimes I have a different bag, I'm gonna forget it some days. I'm not going to use it...  But if it's on my runners… I'm much more likely to just leave it there and make sure that it's there for everything, rather than forget about it… Whereas at least… if it's on the runners, they’re the one thing I always have with me”*​​ |
|  | Ankle | F11: *“Could you attach it around your ankle or something?.. I don't think I’d mind that too much… because you could even wear slightly longer socks to cover it”* |
|  | Thigh | F8: *“Could you wear them like a strap around your quad, your thigh, under your shorts?... f it was around… your thigh, say… the shorts come over it, you mightn’t notice”* |
| Attachment method | Discrete | M2: *“definitely something that is easy. That you can put it on and forget about it… just to be enjoyable and not interfere with what you're doing”* |
|  | Comfortable | F12: “*the main thing would be as long as the sensor is comfortable to run with”* |
|  | Convenient | F4: *“It'd have to be something easily either worn or attached to you”* |
|  | Belt mechanism | F14: *“that’s very easy to wear… So it… ticks lots of boxes”* |
|  | Clip mechanism | M2: *“it has to be very easy to clip to a shoe or… just small clip on the back of the shorts”* |
| Specifications of sensor | Small | M4: *“small enough so it seems easy to have.. in your kit bag if you're going for a run”* |
|  | Lightweight | F9: *“really small and… really light… so you wouldn't really notice you're wearing them”* |
| Good technical features | Infrequent charging of device | F1: *“that it has a good battery life… because that's sometimes… I'd be here working and I’d be like… ‘I never put my watch on charge’... but… I shouldn't be worrying about that too much”* |
|  | Strong Bluetooth connection | F1: *“Strong Bluetooth connection”* |
| Injury-related feedback | Reduce injury risk | M1: *“in terms of looking at injury prevention… I’m at a stage where the injuries are becoming more frequent, and what I can do to avoid them, I’m happy to do it…For most people the thing that stops them from running is injury… if you're running and you're enjoying it, the one thing that's going to stop you is pain…Anything we can learn in terms of telling people how to avoid injuries is best because you keep them involved in it and it’s the same the myself”*  *I’m 53 now, if I can stay around for another 10 years I'll be happy. Ehm, I’d, I’d, I’d prefer not to, to wind up finishing up as a lot of people do when they get back into it in the sort of mid-life, in the mid 40s, they end up finishing by the time they’re 55 or 56.* |
|  | Understand injury mechanisms | M4: *“I’d just be interested in how injuries happen, and tell us how some people could go years without, without an injury running the same way, and then some people have injury after injury, and… what’s the cause of it, and if it can be stopped”* |
|  | Monitor rehabilitation from injury | F9: *“I presume they want to see how they're improving along the way. Like they start with an injury, they want to see improvements. Like you don't want to stay injured forever, so… you'd want to be able to see where you were, like three months ago, to where you are now, to where you should be… when you’ll be able to get back to doing your normal running… You’d like to see… progression as well… Might be interesting… to be able to see that kind of an improvement”* |
|  | Comparison to others | M13 - *“it would be interesting to know… injury reportage… If you could say… ‘in our group of 30-39 year olds, 4% have experienced a tendo-Achilles pain in the last week, and their volume of running was 30-40 kilometres’ then… I'd be like ‘oh that's interesting information’”* |
|  | Advice/Recommendations | F6: *“for example…‘I went for a run today’... And then I come back and I’m like ‘Okay, I feel like I pulled… my hamstring’, and then if I was go onto the app, ‘what should I do in the case of this?’ So maybe if it's like ice, or if it's rest for three days, and then consult your Physio or whatever… like immediate advice to… prevent the injury developing further”* |
|  | Extend running career | M1: *“if I can stay around for another 10 years I'll be happy…I’d prefer not to wind up finishing up, as a lot of people do when they get back into it in the sort of mid-life, in the mid 40s, they end up finishing by the time they’re 55 or 56”* |
| Enhanced data | Performance progressions | M1: *“whatever would improve me as a runner… Everything is about trying to be better than I can be, in my times… it's more about what I could get out of it to see how can I change my running to be better”* |
|  | Optimizing performance | F2: *“performance, optimizing performance… Everyone's looking for the edge”* |
|  | Cadence/Stride information | M12: *I suppose cadence… I suppose stride length as well… if you can see how long you are striding and notice any differences at the start. Are you longer and then as you fatigue, you shorten up, or vice versa. Just to see if there's any differences as a run progresses, does your stride length change”* |
|  | Technique | M13: *“If you're talking about a device that measures a specific… aspect of my running that's not currently measured, like biomechanics or whatever, that would be interesting. And then if it's to just reaffirm some data that I'm collecting already, then that's also cool”* |
|  | Power | M1: *“I don't know much about running power, I haven’t really looked into it too much… but you know, it'd be interesting to see additional information as well … power one would be interesting”* |
|  | Comparison to others | M12: *“if you said ‘oh here’s the 20-29 year old data of 20 runners we have, you are below the mean for your average… distance, but your pace is above average’, that would be interesting, just to see how you stack up in your age range… or demographic if you do it by height and weight… it would be interesting to see how you can stack up again to the mean”* |
