# Supplementary material for: A qualitative examination of the factors affecting the adoption of injury focused wearable technologies in recreational runners
Source: PLoS One. 2022 Jul 6;17(7):e0265475. doi: 10.1371/journal.pone.0265475 (PMC9258862; doi:10.1371/journal.pone.0265475)
Supplement: S5 Table — (DOCX) [file pone.0265475.s005.docx]

**S5 Table: Recommendations for the design of an injury focused smartphone application and wearable sensor**

| **Recommendations for the design of an injury focused smartphone application** | | |
| --- | --- | --- |
| *Sub-theme* | | *Author recommendations* |
| Application design | Quick input session | A single use of an app should take <5 minutes |
|  | Question format | Limit the amount of text entry-type questions. Include multiple choice/tick the box-type questions where possible |
|  | Synced with other applications | Design an app to be compatible with other apps commonly used by runners |
|  | Notification reminders | Send the user notifications to remind them to input data/sync their wearable sensor with their app |
|  | Automatic downloading of data | Where possible, all data collected by a wearable sensor should be automatically transferred to a smartphone app |
| **Recommendations for the design of an injury focused wearable sensor** | | |
| *Sub-theme* | | *Author recommendations* |
| Location | | Design a wearable sensor with the flexibility to allow a range of locations |
| Application method | | Design a wearable sensor with the flexibility to allow a range of application methods |
| Sensor specifications | | Design a small and lightweight sensor which is discrete and comfortable to wear |
