# Supplementary material for: A qualitative examination of the factors affecting the adoption of injury focused wearable technologies in recreational runners
Source: PLoS One. 2022 Jul 6;17(7):e0265475. doi: 10.1371/journal.pone.0265475 (PMC9258862; doi:10.1371/journal.pone.0265475)
Supplement: S1 Appendix — (DOCX) [file pone.0265475.s006.docx]

**S1 Appendix: Pre-focus group questionnaire**

Section 1: Participant Demographics

1. What is your age (in years)? _____________
2. What is your gender?
   1. Male
   2. Female
   3. Non-binary/third gender
   4. Prefer not to say

Section 2: Running Habits & Training History

1. Is running your main sport or activity?
   1. Yes
   2. No
   3. Unsure
2. When did you start running?
   1. Less than 6 months ago
   2. 6-12 months ago
   3. 1-3 years ago
   4. 4-5 years ago
   5. More than 5 years ago
   6. Other. Please specify _______
   7. Unsure
3. How often do you run?
   1. Less than once a week
   2. Once a week
   3. 2-3 times a week
   4. 4-6 times a week
   5. Everyday
   6. Non-consistent routine
   7. Other. Please specify _________
4. Do you take part in organised running events?
   1. Yes
   2. Sometimes
   3. No
   4. Unsure
5. What is your preferred running distance for organised events? Please select all that apply.
   1. Less than 5km
   2. 5km
   3. 10km
   4. Half marathon (21.1km)
   5. Marathon (42.2km)
   6. Ultramarathon (i.e., anything longer than a marathon)
   7. Triathlon
   8. Non-specific
   9. Unsure
   10. Other. Please specify ____________
6. In a typical 12-month period, how many organised running events would you normally take part in?
   1. One event
   2. 2-4 events
   3. 5 events or more
   4. Unsure
   5. Other. Please specify _________
7. What is your average weekly training mileage?
   1. Less than 10km per week
   2. 10-20km per week
   3. 21-30km per week
   4. 31-40km per week
   5. 41-50km per week
   6. More than 50km per week
   7. Unsure
   8. Other. Please specify ____________
8. In which setting do you normally run? Please select the best fit.
   1. Mainly or solely on my own
   2. Mainly or solely with friends, colleagues, or small groups
   3. Mainly or solely with a running club

Section 3: Technology Use

1. What type(s) of running technologies do you use? Please select all that apply.
   1. I do not use any type of running technology
   2. Mobile phone and application (e.g., iPhone & Strava)
   3. GPS watch (e.g., Garmin)
   4. Heart rate monitor
   5. Smartwatch (e.g., Apple Watch)
   6. Wristband activity Tracker (e.g., Fitbit)
   7. Foot pod
   8. Body worn sensor
   9. Other. Please specify ___________

Section 4: Running-Related Injuries

1. Have you had any previous running-related injuries? A running-related injury is any muscle, bone, joint, tendon or ligament pain in the lower back/lower limb(s) that caused you to stop running or restricted your running (either your distance, speed or duration of training)

**AND**

a) That lasted at least 7 days or 3 consecutive scheduled training sessions

**OR**

b) That required you to consult a health care professional

1. Yes, I have had a previous running-related injury
2. No, I have not had a previous running-related injury
3. Thinking of your worst running-related injury, did you miss any training because of it?
   1. I did not miss any training
   2. I missed less than one week
   3. I missed 7-10 days
   4. I missed 2-3 weeks
   5. I missed 4-6 weeks
   6. I missed more than 6 weeks
   7. Other. Please specify _______________
4. How many running-related injuries have you had in the past 12 months?
   1. I have not had a running-related injury in the last 12 months
   2. 1 running-related injury
   3. 2 running-related injuries
   4. 3 running-related injuries
   5. 4 running-related injuries
   6. 5 running-related injuries
   7. More than 5 running-related injuries
5. How important is injury prevention to you for running?
   1. Not at all important
   2. Slightly important
   3. Moderately important
   4. Very important
   5. Extremely important
