# Supplementary material for: A qualitative examination of the factors affecting the adoption of injury focused wearable technologies in recreational runners
Source: PLoS One. 2022 Jul 6;17(7):e0265475. doi: 10.1371/journal.pone.0265475 (PMC9258862; doi:10.1371/journal.pone.0265475)
Supplement: Attachment — Submitted filename: Response to Reviewers.docx [file pone.0265475.s007.docx]

Please find reviewer comments numbered in italics and bold. The authors’ responses are underneath each comment. Each reviewer’s comments begin on a new page. Line numbers are corresponding to the line number in the Word document titled: Revised Manuscript with Track Changes.

Journal requirements:

***When submitting your revision, we need you to address these additional requirements:***

1. ***Please ensure that your manuscript meets PLOS ONE's style requirements, including those for file naming. The PLOS ONE style templates can be found at***
   [***https://journals.plos.org/plosone/s/file?id=wjVg/PLOSOne_formatting_sample_main_body.pdf***](https://journals.plos.org/plosone/s/file?id=wjVg/PLOSOne_formatting_sample_main_body.pdf) ***and*** 
   [***https://journals.plos.org/plosone/s/file?id=ba62/PLOSOne_formatting_sample_title_authors_affiliations.pdf***](https://journals.plos.org/plosone/s/file?id=ba62/PLOSOne_formatting_sample_title_authors_affiliations.pdf).

Adjustments have been made to the font size of headings and sub-headings. Adjustments have been made to the author and affiliations lists. Adjustments have been made to the reference list, where papers with more than six authors are cited “et al.” after the sixth author.

2. ***Please amend your current ethics statement to address the following concerns:
   a) Did participants provide their written or verbal informed consent to participate in this study?
   b) If consent was verbal, please explain i) why written consent was not obtained, ii) how you documented participant consent, and iii) whether the ethics committees/IRB approved this consent procedure.***

Written consent was obtained from participants before completing the pre-focus group questionnaire. This statement in the document has been updated (line 186).

“Prior to taking part in a focus group, participants were required to provide informed written consent and complete a short pre-focus group questionnaire.”

3. ***Thank you for stating the following in the Acknowledgments Section of your manuscript: [The authors would like to thank the focus groups participants for their participation. This publication has emanated from research supported by Science Foundation Ireland (SFI) under Grant Number SFI/12/RC/2289_P2, co-founded by the European Regional Development Fund. We note that you have provided funding information that is not currently declared in your Funding Statement.***

***However, funding information should not appear in the Acknowledgments section or other areas of your manuscript. We will only publish funding information present in the Funding Statement section of the online submission form. Please remove any funding-related text from the manuscript and let us know how you would like to update your Funding Statement.***

***Currently, your Funding Statement reads as follows:***

***[KM was responsible for funding acquisition for this research project. This publication has emanated from research supported by Science Foundation Ireland (SFI) under Grant Number SFI/12/RC/2289_P2, co-founded by the European Regional Development Fund. Funding for the study was received as part of a large-scale, centre-wide funding from Science Foundation Ireland to develop Insight (the national research centre for data analytics:*** [***www.insight-centre.org***](http://www.insight-centre.org/)***). The funders had no role in study design, data collection and analysis, decision to publish, or preparation of the manuscript.]***

***Please include your amended statements within your cover letter; we will change the online submission form on your behalf.***

The funding information has been removed from the Acknowledgements section and does not appear anywhere else in the manuscript. We have updated the funding statement in the cover letter. The funding statement now reads:

[Funding for the study was received as part of a large-scale, centre-wide funding from Science Foundation Ireland to develop Insight (the national research centre for data analytics: [www.insight-centre.org](http://www.insight-centre.org)), under Grant Number SFI/12/RC/2289_P2.]

Reviewer #1
**General comments:**

1. ***The facilitators section read much more fluidly than the barriers sections. I would suggest moving this section first and having the barriers come after. The reason being, that there are much more details in the facilitators sections than the barriers. Additionally, it seems that the facilitators are more impactful than the barriers and since all of them use the equipment, it would be good to know what they like about it first*.**

We agree that it is more appropriate to present the ‘facilitators’ findings first. This has been changed in the manuscript. Appropriate changes were made to reflect this throughout the document (e.g., relabelling tables).

2. ***Generally, it is not clear what sort of “device” the runners are discussing in terms of monitoring injury. Is this just a hypothetical app or something that they already know about/use? It is also unclear who the “manufacturers” are. I would guess that most app developers do not consider themselves as manufacturers*.**

During the focus groups, an example of a hypothetical wearable sensor and compatible smartphone application was described to participants to encourage discussion regarding their perceived use of injury focused technologies. Participants were probed to discuss this in relation to their experience with other running- and/or health-related applications, as well as their perceived use of a new injury focused technology (i.e., the hypothetical device described). The following statement has been added to the ‘Methods’ section to clarify the type of device:

“To aid discussion of injury focused technologies, it was suggested that a hypothetical smartphone application (app) could collect both sensed data (from a sensor) and data that users would be required to input manually (potentially before and/or after a run). Participants were probed to discuss this in relation to their experience with other running- and/or health-related apps, as well as their perceived use of a new injury focused technology (i.e., the hypothetical technology described)” (lines 208-214).

The phraseology “manufacturer” has been replaced with “technology developer”. In the case of “technology developers”, we are referring to those who design and develop wearable devices or sensors. In the case of “app developers”, we are referring to those who develop smartphone applications. We have been mindful of terminology used here as elements of the study relate to the physical sensor itself, which would be outside the remit of many app developers.

3. ***No need to use decimals among the percentages. Does not add to anything by specifying the decimal.***

This has been updated in throughout.

4. ***A simplified table of the most important factors (ranked) would be useful. And maybe a table of recommendations for app/device developers. It would be good to know how many metrics should be included in a device/app. There seems to be some categories (i.e. metrics, wearability, usability etc) that could be explained a bit better…again, potentially with a table or figure of some kind***

Table 2 (Running-related injury risk factors perceived as important to monitor using wearable technology devices) has been simplified to include metrics only discussed by at least 3 participants (for simplicity); however the full coding framework is included in the supplementary material for readers interested in all responses. This is referenced in-text. With regard to the ranking of metrics discussed, we have ordered the table by how often each theme was discussed during focus groups.

A table has been developed which highlights our recommendations for the design of an injury focused smartphone application and wearable sensor. It is included in the Supplementary Material (F), and referenced in the manuscript (line 918). However, we feel we cannot offer a recommendation for the number of metrics that should be included in an application as this was not discussed during the focus groups.

**Supplementary Material F: Recommendations for the design of an injury focused smartphone application and wearable sensor**

| **Recommendations for the design of an injury focused smartphone application** | | |
| --- | --- | --- |
| *Sub-theme* | | *Author recommendations* |
| Application design | Quick input session | A single use of an app should take <5 minutes |
|  | Question format | Limit the amount of text entry-type questions. Include multiple choice/tick the box-type questions where possible |
|  | Synced with other applications | Design an app to be compatible with other apps commonly used by runners |
|  | Notification reminders | Send the user notifications to remind them to input data/sync their wearable sensor with their app |
|  | Automatic downloading of data | Where possible, all data collected by a wearable sensor shold be automatically transferred to a smartphone app |
| **Recommendations for the design of an injury focused wearable sensor** | | |
| *Sub-theme* | | *Author recommendations* |
| Location | | Design a wearable sensor with the flexibility to allow a range of locations |
| Application method | | Design a wearable sensor with the flexibility to allow a range of application methods |
| Sensor specifications | | Design a small and lightweight sensor which is discrete and comfortable to wear |

5. ***The discussion section could be improved with some further headings to structure and enhance flow. There is also a lot of repetition of study results in the discussion section. Suggest revising whole section to improve clarity/flow. The superfluous word-padding can be clarified by writing in an active voice and removing some of the nominalisations that are found throughout the whole paper.***

We appreciate your comment regarding further sub-headings and agree that this would help to enhance the flow of the paper. Unfortunately, with the journal requirements, a maximum of 3 sub-headings is permitted, limiting us from adding further sub-headings in the results/discussion sections.

We have reviewed the discussion section to improve clarity/flow. We have removed superfluous word padding, written it in the active voice and premised and contextualised the results in the discussion, allowing the reader to follow our points more easily.

**Specific Comments:**
Abstract:

1. ***Line 20: the word “use” is presented 4 times in this sentence…rephrase to improve flow.***

This sentence has been re-phrased and now reads:

“Understanding the perceived efficacy and ease of use of technologies will influence initial adoption and sustained utilization ” (lines 43-44).

2. ***Line 32: Unclear what you mean that the attachment method and app are barriers and facilitators.***

This sentence has been rephrased to improve clarity, and now reads:

“The location and method of attachment of a wearable device, the design of a smartphone application, and receiving useful injury-related feedback will affect recreational runners’ adoption of injury focused technologies.” (lines 53-56).

Introduction:

1. ***Line 44-46: by “accurate and objective” does this mean valid and reliable?***

This line has been re-written to improve clarity, and now reads:

“Advancements in wearable technologies have made it possible for the early detection of illness/injuries by continually monitoring individuals.” (lines 77-79).

2. ***Line 49: specify “data”***

‘Data’ has been specified in this sentence, with the line now reading:

“Primarily, wearable devices in this market function to collect global positioning system (GPS) data and information on running technique to provide summary reports to assist running performance.” (lines 82-84).

3. ***Line 62 - 63: rephrase to active voice improve clarity “Therefore, it is crucial to identify the metrics rec. runners perceive as important for monitoring injury risk and adopting injury-prevention technology”.***

This sentence has been re-written to improve clarity, and now reads :

“Therefore, it is crucial to identify the metrics recreational runners perceive as important for monitoring injury risk and adopting injury focused technology.” (lines 104-105).

4. ***Line 69: remove sentence as it is does not add to the argument and comes across as arrogant.***

This sentence has been removed from the manuscript.

5. ***Line 74 - 75: rephrase to active voice to improve clarity (remove nominalised verbs)***

This line has been re-written to improve clarity and now reads:

“Therefore, the aim of this study was to examine the factors that affect injury focused technology adoption in recreational runners, to identify the metrics perceived as important to monitor for RRI risk, and to identify the perceived barriers and facilitators to the utilization of injury focused technologies.” (lines 115-118).

Methods:

1. ***Did you use the COREQ guidelines (or similar?) for reporting your study? State this at the front of your methods.***

We used ‘The Standards for Reporting Qualitative Research’ (O’Brien et al., 2014) for reporting our findings. A sentence stating this is now presented in the opening paragraph of the Methods section and reads:

“The Standards for Reporting Qualitative Research (36) (Supplementary Material A) were adhered to” (lines 152-153).

2. ***Line 104: spell out the number “6”***

This has been changed in the manuscript (along with the use of other numbers between 0-9), and the sentence now reads:

“Eligible participants had to be aged between 20 and 60 years and meet Mulvad and colleagues’ (37) definition of a recreational runner; someone running at least once per week, for at least six months.” (line 160-163).

3. ***Lines 107 – 109: rephrase to active voice to improve clarity.***

This sentence has been re-written to improve clarity, and now reads:

“A pilot study was conducted to educate and train the primary author in efficient focus group moderation techniques, and in the use of an analytical framework for analysing qualitative data.” (line 173-175).

4. ***Line 121: spell out numbers zero to nine…correct throughout***

This has been corrected throughout the manuscript. This line now reads:

“A RRI was defined as any musculoskeletal pain in the lower back/lower limbs that causes a restriction to or stoppage of running for at least seven days or three consecutive scheduled sessions, or that causes a runner to consult a healthcare professional.” (line 190).

5. ***Lines 139-141: Please clarify this process…do not understand what you mean by success and success of each discussion topic.***

These statements have been re-written to improve clarity, and now read:

“A reflective and iterative approach was taken with regard to focus group moderation and the content of the focus group schedule. Additional probes were included in the focus group schedule and adjustments to moderation techniques, (e.g., ensuring equal speaking opportunities for all participants) were made during this data collection phase.” (line 219-221).

Previously, the line read: Following each focus group, its success and the success of each discussion topic were considered by the research team, with any potential changes being discussed and agreed upon, prior to execution.

6. **Line 165: was reliability of coding reported?**

Quality criteria utilised in quantitative research (reliability, validity etc.) are not appropriate in qualitative research and thus, quality and rigor of findings can be determined through trustworthiness (Korstjens and Moser, 2018). Trustworthiness includes the concepts of credibility, transferability, dependability, confirmability and reflexivity (Korstjens and Moser, 2018). The trustworthiness of the current findings already is detailed in the manuscript: section 3.6 - Trustworthiness. The authors utilised a number of approaches to enhance trustworthiness, including a critical friend approach, investigator triangulation, thick description and the demonstration of multi-vocality and diverse contribution from participants.

*Korstjens I, Moser A. Series: Practical guidance to qualitative research. Part 4: Trustworthiness and publishing. European Journal of General Practice. 2018; 24(1):120-24.*

7. **Line 174: if reliability is not reported in the results, could be stated here.**

This comment has been addressed in detail above. In addition, detail of the trustworthiness of the data is evident in section 3.6.

Results:

1. **Table 1 is difficult to follow…maybe a graph would be easier? I’m not sure I have a suggestion to improve, it’s just hard to follow.**

We appreciate your comment regarding the formatting of Table 1 and understand that there is a lot of information presented here. We have looked again at how best to present information contained in Table 1. We generated a few different versions with colleagues and all colleagues invariably felt that the current version of Table 1 (with some minor changes to the text) is the clearest, and that it was not ‘too difficult’ to follow. Therefore, we have decided to keep the table in this format. We have also added some additional information from the questionnaire to the table in response to other comments.

2. ***Line 245: remove contraction “haven’t”…not part of the quote so should be spelled out.***

This line has since been removed from the manuscript, following the refinement of the sub-themes presented both in Table 1 and in text of the Results section.

3. **Lines 255 and 258: repetitive statements here…one is the quote and one is the authors statement. Rephrase these sentences to improve flow**

These sentences has been restructured, and now reads:

“The terrain on which people ran was commonly perceived as a potential risk factor for injury. There was generally a lack of consensus between participants as to which surfaces posed the greatest risk, despite one participant (M2) describing this metric as “really important to take into consideration”. (lines 412-414).

4. ***Line 262: include [shoes] next to the participants use of word “runners” as it could be confusing to the reader and interpreted as a person rather than the shoes.***

This line now reads:

“One participant (M7) described their interest in understanding *“how more injury prone you are, dependent on both the age of the runners* [shoes] *you use, and the different brands of runner [shoe] you use”*.” (line 420).

5. ***Line 295: The category “useless feedback” seems a bit harsh and inaccurate considering the subthemes. Too much feedback might not be useless and the feedback delivery subtheme does not fit under the main theme…suggest a main theme along the lines of “ineffective information” or “poor knowledge translation”.***

We have renamied this category “ineffective feedback” (previously “useless feedback”). This has been changed throughout the manuscript.

6. **Line 301-311: This section is not very clear. It would be more beneficial to describe specific apps (without naming??) that participants discussed and then talk about those barriers. Apps that I would expect most runners use, especially if they are paired to a smart watch, often collect the data automatically. So I guess I am not aware of what functions apps have where a lot of repetitive questions are asked….Furthermore, did you ask about what types of equipment they use? This would be a good thing to report in a participant characteristics table. Then the reader could make some judgements about the equipment discussed in this section (and I’m assuming further sections).**

When prompted, participants considered that this hypothetical app would collect both sensed data (from a sensor) and data that they would be required to input manually (potentially before and/or after a run). This section has been clarified and the context in which participants discussed this section has been included. This section now reads:

“Participants discussed how the application design could act as a barrier to injury focused technology use, with a high demand on the user serving as a barrier. This was discussed in relation to participants’ previous experience with other health- and running-related applications, as well as their perceived behaviour for engaging with a hypothetical injury focused application. Participants considered that this hypothetical app would collect both sensed data (from a sensor) and data that they would be required to input manually (potentially before and/or after a run). A large time requirement for inputting data was identified as a potential barrier to technology use, with M5 suggesting: “*realistically if it'll be any more than a couple minutes and people get bored putting in the data”.* Participants discussed their tolerance and willingness to engage with such an application, and it was identified that five minutes was deemed the maximum amount of time runners were willing to spend using an application - M6 - “*five minutes probably would be my max”.* From previous experience, a requirement to respond to a high quantity of questions (more than four questions) was described as *“onerous”* (F8) therefore identifying a further potential barrier. Questions deemed as irrelevant and repetitive were also described as a barrier with one participant indicating: “It just gets a bit tedious… basically it’d [wearable wrist-based device monitoring sleep and recovery] ask you loads of questions, and it's like the same questions over and over” (M11). (lines 562-585).

Additionally, further information from the pre-focus questionnaire, including device use, has been added to Table 1: Participant demographics and characteristics.

7. **Line 321: should be “non-preferred”**

This has been corrected in the manuscript, with the line now reading:

“Differences in the non-preferred locations of a wearable device were evident, with some describing the lower back as an undesirable location as it was perceived as uncomfortable or that it may “rub against your skin and get a bit sore” (M8).” (line 595).

8. **Line 326: second time using the phrase “general consensus” in this paragraph. Need to rephrase some of the sentences to improve flow.**

This has been changed and the line now reads:

“Variance in the opinion made it difficult to determine any specific location as a barrier to use; however, the majority agreed that locations perceived as uncomfortable, one’s which resulted in excessive movement of the device, or were “very obvious” (F11) to others would result in reduced compliance, and therefore act as barriers to usage.” (line 600).

Discussion:

1. ***Lines 441 – 443: Unclear what you mean here.***

This has been clarified in the manuscript, and the line now reads:

“Overtraining, as reported by participants of the current study also maps to the biomechanical model of injury, whereby loading of tissues beyond their adaptive capability, combined with insufficient recovery, results in injury (52,54).” (lines 674-698).

2. ***Line 454: new paragraph, have switched topics from terrain to cross-training***

Paragraph inserted

3. ***Line 461: new paragraph, have switched topics from concurrent sports to running technique.***

Paragraph inserted

4. ***Line 476: explain what you mean by ‘bilateral asymmetry’***

The term ‘bilateral asymmetry’ has been explained earlier in the document, at its first mention. This line reads:

“With running technique, runners suggested that foot strike technique, bilateral asymmetries (i.e., a difference between left and right lower limbs) and cadence may be factors that influence the onset of RRI’s.” (line 388).

An explanation has also been included in the line related to this comment. This line now reads: “Bilateral asymmetry, which relates to differences between the left and right lower limbs, has been suggested as a risk factor for RRI’s based on the premise that because one leg is subjected to more loading, it is predisposed to injury (70,71) (lines 792-794).

5. ***Line 481: new paragraph***

Paragraph inserted

6. ***Line 492-93: please clarify this sentence. Hard to follow your argument here***

This sentence has been clarified, and now reads:

“Runners have also failed to acknowledge the importance of a previous injury as a risk factor for injury in an earlier qualitative study (52).” (lines 820-821).

7. ***Line 494: who should be monitoring the runner’s injury history?***

This line now reads:

“While this may reflect a sense of being ‘unable to change’ the occurrence of having a previous injury, it clearly should be taken into account (via an application) when monitoring for the purpose of preventing re-injury.” (line 823).

8. ***Line 499: improve sentence structure so that you do not end the sentence with a preposition***

This sentence has been restructured, and now reads:

“Different from an injury, in which a runner is forced to reduce or stop training for a period of time (38), our participants’ perception of ‘niggles’ is similar to previous research where runners described ‘complaints’ as ‘small pains’ with which they can continue to run (28).” (line 809).

9. ***Lines 513-514: unclear what you mean by “general” and “specific” RRIs***

We have re-written this statement to improve clarity, and provided examples of ‘specific’ RRIs. It now reads:

“While there are ‘conflicting’ (63) and ‘inconsistent’ (64) results for a relationship with RRI’s in general, high peak and rates of loading have been found to contribute to the development of *specific* RRI’s, such as bony and soft tissue injuries (75) and stress fractures (76).” (lines 837-840).

10. ***Line 516: remove the word “both”***

“Both” has been removed from the sentence. It now reads:

“These findings expand on the current evidence and report new findings in relation to the metrics deemed important by runners for monitoring RRI risk when using wearable technologies.” (lines 865-867).

11. ***Line 518: remove the word “clearly”***

“Clearly” has been removed from the sentence, and it now reads:

“Firstly, injury focused technologies should monitor risk factors that are deemed important by runners, where evidence-based research supports their relevance (e.g. excessive loading and inadequate recovery).” (line 867).

12. ***Line 519 – 524: I struggle to agree with your statement here. You have just spent a great deal of effort identifying that there is not a lot of evidence-based support in favour of this study’s participant’s ideas/thoughts. You then follow on to leave it up to app developers to decide what should be provided. This seems counter-productive for the purpose of this study. It would make a stronger argument to just state that if manufacturers decide to develop products that focus on the runner’s wants/needs…then do XYZ…and be specific rather than just state “include factors supported by evidence-based research”…State that apps need to be designed that include specific components, and that more research is needed to further support the factors that are not currently evidence based, but are deemed important by runners.***

As per our statement “Firstly, injury focused technologies should monitor risk factors that are deemed important by runners, where evidence-based research supports their relevance (e.g. excessive loading and inadequate recovery)” (lines 867-869);

The challenge faced by app developers is what should they do when:

1. users do not see the importance of capturing certain information (e.g. previous injury), when research evidence indicates it is a risk factor for RRIs

and

2. users believe a metric should be monitored when there research suggests it is not a risk factor (e.g. terrain and foot strike technique)

From an evidence-based approach to injury prevention, factors increasing the risk of injury should be monitored. The problem is that if users see them as irrelevant, it is possible their inclusion will decrease the likelihood of the app being adopted (point 1). For example, users may believe that inputting such information will take too much time or that the app developers do not understand the causes of RRIs.

Similarly, if app developers do not include a metric that users believe is important (point 2) because the app developers recognise that the research evidence suggests it is not a risk factor for RRIs, then they risk users believing that the app developers do not understand the causes of RRIs.

In the case of point 1, we believe the solution to this is to educate runners.

In the case of point 2, we believe that app developers need to decide on a balance between user perceived requirements and the additional time required for user input in capturing this data. We hope the altered text clarifies this important discussion point.

A comment about the need for further research has also been added to this paragraph. This now reads:

“Firstly, injury focused technologies should monitor risk factors that are deemed important by runners, where evidence-based research supports their relevance (e.g. excessive loading and inadequate recovery). The challenge for app developers is whether to include metrics that monitor risk factors that are: (i) not deemed important by runners, but research does support their relevance (e.g. previous injury), or/and (ii) that are deemed important by runners, but current research does not support their relevance (e.g. terrain and foot strike technique). In the case of the first point, the authors would strongly advocate for including factors supported by evidence-based research (e.g., previous injury) with efforts made by app developers to educate runners in potentially valuable metrics. This is important in order to improve the perceived usefulness of devices (18,19,20). In the case of the second point, the inclusion of these metrics (e.g., terrain and foot strike technique) may be useful if they encourage technology adoption and uptake. This must be balanced against the additional time needed by the user to input this data, which itself may be a barrier to app adoption and continued utilization (discussed below) ” (line 867-880).

13. ***Line 533: difficult to follow with double negative statement***

Yes, when we first wrote this we saw the challenge associated with the double negative. The problem is that it is not possible to change it to a single negative or a double positive statement, because of the nature of the argument. We have attempted to rephrase it somewhat in order to improve clarity. The line now reads:

“Also, a lack of research evidence (or mixed evidence) to support a relationship between a metric and an increased risk of a RRI does not necessarily indicate that there is no relationship, but may reflect the inability of current research to effectively examine the relationship.” (lines 880-883).

14. ***Lines 542 – 545: long topic sentence. Improve for clarity and flow.***

This sentence has been re-written to improve clarity. It now reads:

“The above findings are also important to coaches and clinicians in developing intervention strategies for injury prevention, where uptake and adherence by runners is improved when runner perception aligns with intervention design (20).” (lines 910-913).

15. ***~~Lines 545- 550~~: sentences here are not supported with the topic sentence…need to create a new paragraph for these and add content for first sentence.***

A new paragraph has been created for this section. The paragraph now reads:

“These findings also raise the question about how runners form their opinions that a metric is a risk factor for RRIs, when the research evidence would suggest it is not a risk factor. These perceptions may be due to widely available information on popular running websites. There are many examples of low cadence (84), heel-striking (85), and harder terrains such as concrete (86) being described as risk factors for RRI’s on websites, despite a lack of supporting scientific evidence. Clearly there is a need for the scientific community to better educate runners.” (lines 842-863).

16. ***Line 573: please clarify “device location” do you mean “wear-location” as explained above? Be consistent with terms here.***

This term has been changed, and the line now reads:

“However, it has been suggested that athletes of varying sports (e.g., volleyball) may find wear-location to be a potential barrier to use (93).” (line 956)

17. ***Line 575: improve sentence clarity “to be seen to be….” Hard to follow***

This statement has been re-written to improve clarity, and now reads:

“Additionally, some participants suggested that they would not like a device to be noticeable or obvious to others, as they did not want others to know they would be self-monitoring. This finding has not previously been identified in recreational runners, but has been found in relation to health based monitoring with wearables (94).” (lines 956-960).

18. ***Line 612: it is still unclear what “useless information” means***

We have re-phrased this statement in order to improve clarity. The line now reads:

“It is well understood that maintaining user interest (95,100) and receiving useful and accurate data (90,99) can facilitate the use of wearable technologies; while the collection and reporting of inaccurate data and irrelevant information have been suggested as barriers to use of physical activity tracking technologies (26,88,89,90,91,93).” (line 1001)

19. ***Line 613 – 616: this argument is not well developed. I think this could be improved by explaining which components of the models you are trying to link together here…maybe in a new paragraph and some supporting evidence.***

We agree that additional information is required to develop this argument, and so we have included some more detail here. We have also inserted a new paragraph. Previously, this argument was written as:

“In line with the TRIPP model for enhancing injury prevention practices (21), and considering the TAM (19) and UTAUT (20)  models for predicting technology usage, runners are more likely to use technologies if they provide runners with an understanding of the mechanisms of injury, and prove to be useful in preventing injury.”

This new paragraph now reads:

“In line with the TRIPP model (Stage 5: intervention context to inform implementation strategies), the successful implementation of injury prevention practices will be determined by, among other factors, the likelihood of its uptake (20). In order to improve uptake, researchers (and those issuing injury prevention programmes) must understand why injury prevention practices may or may not be adopted by the target population and provide confidence that adoption of the intervention will reduce the likelihood of injury (20). Additionally, the TAM (18) and UTUAT (19) models suggest that technologies are more likely to be adopted if they are perceived as both useful and easy to use. Receiving relevant feedback is one way of improving the perceived usefulness of a device, as suggested by participants of the current study” (lines 1003- 1012).

20. ***Line 623: improve sentence flow “qualitative informative insight” is a lot of words when you mean “perceptions”***

The sentence now reads:

“The current study provides insight into the factors affecting the adoption of injury focused technologies in recreational runners.” (line 1020-1021).

21. ***Line 627: the comparative analysis is a strength of rigour during data analysis…be specific about the strengths of your study***

This has been specified and the sentence now reads:

“Employing constant comparative analysis throughout the data collection phase, improved the study’s methodological rigour.” (lines 1022-1024).

22. ***Line 645 – 646: this is a strong statement that should be presented near the front of the discussion.***

Thank you for this comment. We have presented this statement at the beginning of the discussion:

“Overtraining, training-related, and individual-related risk factors are essential metrics that need to be monitored using wearable technologies for RRI risk.” (line 630-631).

Reviewer #2:

**Major Comments:**

1. ***Supplementary quotes to build trustworthiness: I appreciate there are word count challenges with qualitative results, however I did find that many of the quotes included in the results section were very short, sometimes even only 1-2 words. I would suggest including a supplementary table that includes additional quotes representing each sub-theme. This will grow the readers trust in your findings. The way the results are currently presented the data (quotes) rely heavily on the authors text to be interpreted.
   *** Note, that now I am seeing that there are some further data included in the Supplementary material E- this was not referenced when presenting the results, this needs to be flagged for the reading. I would suggest minimum 3 quotes per subtheme, most only appear to have 1.***

Additional quotes have been added throughout the results section. We recognise that some of the quotes rely on our own text to be interpreted; this was deliberate in order to reduce the overlength of the paper and to aid understanding. Additionally, the supplementary quotes table has been referenced in the document (line 279), thereby pointing the reader to the additional quotes that you have requested.

2. ***Further refinement of themes: In table 2,3, and 4 I found it interesting that the authors choose to include the number of participants who discussed each theme. While this is good for transparency it also made me think that some of the results needed further consideration – for example subthemes in table 2 high stress and weather only were mentioned by 1 participant. Does that truly warrant them to be their own subtheme? Perhaps there needs to be further refinement of subthemes? Similarly, I noticed this in table 3 and 4 with regards to barriers and facilitators. The authors mentioned how there are many factors that can be both barriers and facilitators. I’m wondering if there is a better way to present this data showing how certain factors can be both. Maybe you could consider combining these tables/themes and I think this could be more meaningful for the reader and future work. For example, instead of having 2 separate core categories as “useless feedback received’ and “receiving useful feedback” consider 1 core category “feedback” then show the useful and useless side by side in same table.***

Tables 2-4 have been refined to only include sub-themes discussed by at least 3 participants. However, the full coding frameworks have been retained in the Supplementary Material.

Tables 3-4 have been merged into one table (Table 3, line 464), thereby presenting the barriers and facilitators together. However, the written text relating to the barriers and facilitators are presented in separate subsections in the results for clarity (and because the participants discussed these separately).

3. ***Discussion needs to be more focused and concise. There is a lot of repeating of the results in the discussion, I would try to limit this. For example, the discussion repeats results on what factors runners believe are important for monitoring. It would be nice if authors can instead of repeating results, maybe suggest how these factors can actually be monitored? Or what research has been previously been done to monitor these risk factors. For example, I know my Garmin watch does track some of these overtraining themes. (line 550).
   Also there seems to be a lot of discussion about runners perceived risk factors for injury compared to what the evidence says for risk factors for injury. This seems to be a bit off topic from the original aims of the study. Suggest shortening some of these paragraphs and make sure it stays focused on main purpose of this study.
   The concluding paragraph is very strong! Use this to guide the discussion – expand on ideas brought up here.***

We have removed some of the results from the discussion as requested. However, in order to contextualise the discussion and allow the reader to follow our discussion more easily, there is a requirement to detail some of the results.

The aim of this study was not to say how specifically these metrics can be monitored, and we did not ask participants to comment in this. Instead, in line with our aims, we have discussed our findings in comparison to what previous evidence suggests are risk factors for injury, and provide detail on *what* metrics should be monitored. We have not provided a recommendation of how to capture these metrics, due to the complex nature of some of those identified (for example, how to monitor overtraining) and because we did not ask the participants to comment on this.

Regarding “there seems to be a lot of discussion about runners' perceived risk factors for injury compared to what the evidence says for risk factors for injury”; we feel this is important because this directly impacts whether a risk factor should be included in an app or not. The importance of this relationship between perceived risk factors and actual (evidence based) risk factors is discussed in detail in lines 663-913, and is an implicit element of the main aims. In addition, both reviewers’ 1 and 3 comment positively on this comparison.

4. ***Looking at the supplementary files of the pre-focus group questionnaire, there is a lot of valuable descriptive data that was collected that could be used be better describe the sample for the reader. This could be included in Table 1 and/or at the start of the results. Details such as do you take part in organized running events, preferred running distance, average training mileages, social running context, etc.***

We have added further detail to Table 1 (Participant demographics and characteristics). Details regarding technology use have been included to add context to the topics discussed later.

Introduction:

1. ***Line 51-52: the authors state there is a high rate of RRIs – perhaps a sentence or two and reference that provides some details about RRI incidence in recreational runners.***

Further information has been included, and the line now reads:

“However, despite the high incidence of running related injuries (RRIs), recently reported at 40% (12) and 46% (13), and the popular use of wearable devices to manage other illnesses and injuries (14,15,16)” (lines 86-87).

2. ***Line 53-54: While the authors state wearables have not been investigated for usefulness in injury prevention, if they could touch on what other injury prevention strategies that have been researched – and the fact that they are not very successful overall – would strengthen the introduction.***

We agree that some information on other injury prevention inventions would strengthen the introduction. A statement has been added to the introduction, with the line reading:

“However, despite the high incidence of running related injuries (RRIs) (12,13), and the popular use of wearable devices to manage other illnesses and injuries (14,15,16), there is a dearth of research investigating the perceived usefulness of injury focused wearable technologies in runners. Additionally, no effective injury prevention programmes for reducing RRIs have been identified in the literature thus far (17). It has been hypothesized that this is due to previous injury prevention programmes focusing on reducing the impact of a single risk factor, when the cause of RRIs is multifactorial (17). (lines 85-92).

Methods:

1. ***Lines 101-104: suggest including more clear inclusion criteria in the methods and details about how these recreational runners were purposively sampled – i.e. running clubs, online, social networks? I noticed in the limitations it mentioned that running volume, etc was not considered in the sampling strategy. This could be mentioned here. In addition, I would move the details about actual number recruited and their demographics to the beginning of the results section.***

We agree. Further details have been included, and this section now reads:

“A purposive sample of 27 adult recreational runners were recruited. Local running clubs were contacted via email and asked to distribute research information and contact details to potential participants. Those interested then contacted the researchers. Eligible participants had to be aged between 20 and 60 years and meet Mulvad and colleagues’ (35) definition of a recreational runner: someone running at least once per week, for at least six months. A minimum/maximum running volume or the use of wearable technologies was not included in the inclusion criteria.” (line 158-164)

Additionally, participants’ demographic information has been removed from the Methods section and included in the beginning of the Results instead.

2. ***Line 107-113: Pilot study results. It is not clear if the focus groups/ analysis here were included in the main study results or if this was only used as training for the primary author. Where modifications made to the workshop guide based on these? If so, please provide some examples.***

Further details have been included in this section (line 173-182).

“A pilot study was conducted to educate and train the primary author in efficient focus group moderation techniques, and in the use of an analytical framework for analysing qualitative data. The results of the pilot study were not included in the main study results. The focus group schedule was updated throughout this pilot phase to include more open-ended questions and additional probes to include all participants in the discussion (e.g., “What do you think M#, have you any thoughts on that?”). Four male and five female recreational runners were recruited as a convenience sample, aged 25.1 years ± 2.2 years. Three separate pilot study focus groups were facilitated by the primary author, each taking place via remote video conferencing software (Zoom, version 5.7.0) and lasted 39.1 minutes ± 5.4 minutes.”

**Results:
See major comments above.**

**Discussion:
See major comments above.**

1. ***Line 424-426- It sounds like the findings about perceived ease of use and perceived usefulness are already known from previous theories, based on the way this sentence is phrased. Consider explaining how your findings can be interpreted or enhanced using the models.***

We have added some additional details and content to this section regarding these models, which we believe enhances our findings. This section now reads:

“ The findings of the current study are similar to the Technology Acceptance Model (TAM) (18) and the Unified Theory of Acceptance and Use of Technology (UTAUT) (19), which suggest that individuals’ use of technology will be influenced by a number of factors. Such factors include: the perceived ease of use and perceived usefulness of a device/app (as suggested by the TAM [18]), and the effort expectancy (which is preceded by ease of use, perceived ease of use, and complexity), performance expectancy (i.e., the degree to which an individual believes a technology will help to improve their injury risk [in the case of the current study]), and behavioural intention to use a device/app (as suggested by the UTAUT [19]). Our findings map to these models and as discussed below, we found that perceived usefulness and/or performance expectancy (i.e., the metrics perceived as important for monitoring RRI risk and feedback received), and perceived ease of use and/or effort expectancy (i.e., difficulty/ease of use) will influence recreational runners’ behavioural intentions to use injury focused wearable technologies. App developers (those developing smartphone applications) and technology developers (those designing wearable devices/sensors) can draw upon these theories and the findings of the current study to design and manufacture injury focused wearable technologies suitable for use by recreational runners.” (lines 634-660)

Table 1:

1. ***Suggest having n=27 in Title at top of table. Then in the 3 lines with missing data indicate with symbol and footnote for number of respondents.***

Table 1 has been updated in the document.

2. ***Did no participants have more than 2 RRI’s or is this column 2 or more RRIs?***

No participant reported having more than 2 RRIs in the previous 12 months (Table 1).

**Table 2 & 3 & 4:
See major comments.**

Supplementary material C:
***Appears to be a duplicate of tables? Assuming this is the coding matrix that was inductively generated from the data, it is already in the paper?***

Yes, there was duplication between the tables in the supplementary material and the main paper. This was because the supplementary table contained all of the codes (as a stand-alone document), while the table in the main text aimed to capture only the most prevalent codes.

In order to address 2 reviewer’s comments, Table 2 in the main text now only contains sub-themes identified by at least 3 participants, thereby reducing the content of Table 2 in the main text. We feel it is now more appropriate to keep the full coding framework in the Supplementary Material to highlight all codes (as a stand-alone reference table) that were generated from the focus group transcripts.

Reviewer #3:

1. ***The synthesis of the interview gives a good overview of participants opinion, and I think authors should highlight that training load related data and recovery data reported are in line with the current knowledge about RRI.***

A statement, with references, has been added to the discussion, reading:

“Overtraining, consisting of excessive loading and inadequate recovery, was perceived as a leading risk factor for the development of RRI’s in the current study, in line with current knowledge about RRIs (51) and similar to the perceptions of recreational runners in previous studies (28,53).” (line 667).

2. ***However, I am a bit surprised to see runners running less than 1/week included, this should be justified*.**

Apologies, this was an error on our behalf. No one was running less than 1/week. This has been rectified in the Table 1 (line 282)

3. ***The discussion is good and meaningful, and as stated lines 542-546, I suggest to write in the conclusion/perspectives that it would be interesting to compare those data to those collected in a sample of coaches/clinician.***

Thank you for your comment here. We agree that it would indeed be very interesting to gather the thoughts of clinicians and running coaches on the metrics perceived as important for monitoring RRI risk. We have included a statement in the concluding paragraph of the ‘metrics’ section (5.1 Metrics important for monitoring RRI risk), which reads:

- “In addition, future research should include clinicians and running coaches, as their thoughts and opinions may yield further insight into the metrics deemed important for monitoring RRI risk” (lines 904-906)

We feel it is more appropriate to include this statement in this section of the discussion, rather than in the final conclusion of the manuscript, as it is on-topic to this section and improves the flow of the section.

4. ***Finally, I feel that the section about feedbacks could be more meaningfull: would participant be willing to get a “ global” RRI score, or specific advices , ie : load is too much, pay attention to sleep, running biomechanics is altered…***

This is an excellent point but was something not discussed with or by the participants. However we will certainly include it in future research. This has been added to the limitations of the research, in a statement reading:

“Finally, there was potential scope for additional probing during the data collection phase, with some topics requiring further exploration and explanation. For example, runners’ perceptions of including an ‘overall RRI risk score’ into wearable technologies was not examined in the current study. This additional probing and line of questioning may potentially yield further information; an observation that should be considered by future researchers” (lines 1049-1054).
